# A Novel Indoline Derivative Ameliorates Diabesity-Induced Chronic Kidney Disease by Reducing Metabolic Abnormalities

**DOI:** 10.3389/fendo.2020.00091

**Published:** 2020-03-10

**Authors:** Anna Permyakova, Asaad Gammal, Liad Hinden, Michal Weitman, Marta Weinstock, Joseph Tam

**Affiliations:** ^1^Faculty of Medicine, School of Pharmacy, Institute for Drug Research, The Hebrew University of Jerusalem, Jerusalem, Israel; ^2^Department of Chemistry, Bar Ilan University, Ramat Gan, Israel

**Keywords:** obesity, diabetes, chronic kidney disease, indoline derivative, insulin resistance, fatty liver

## Abstract

Both diabetes and obesity (diabesity) contribute significantly to the development of chronic kidney disease (CKD). In search of new remedies to reverse or arrest the progression of CKD, we examined the therapeutic potential of a novel compound, AN1284, in a mouse model of CKD induced by type 2 diabetes with obesity. Six-week-old BKS Cg-Dock 7^m^+/+ Lepr^db^/J mice with type 2 diabetes and obesity were treated with AN1284 (2.5 or 5 mg kg^−1^ per day) via micro-osmotic pumps implanted subcutaneously for 3 months. Measures included renal, pancreatic, and liver assessment as well as energy utilization. AN1284 improved kidney function in BSK-*db/db* animals by reducing albumin and creatinine and preventing renal inflammation and morphological changes. The treatment was associated with weight loss, decreased body fat mass, increased utilization of body fat toward energy, preservation of insulin sensitivity and pancreatic β cell mass, and reduction of dyslipidemia, hepatic steatosis, and liver injury. This indoline derivative protected the kidney from the deleterious effects of hyperglycemia by ameliorating the metabolic abnormalities of diabetes. It could have therapeutic potential for preventing CKD in human subjects with diabesity.

## Introduction

Over the past decades, diabetes and obesity have evolved into global epidemics, affecting social groups and ages in both developed and developing countries. Diabesity, a new term used to describe the co-existence of these two conditions (1), results in the death of over a million people worldwide each year, and its global prevalence exceeds 347 million. It is also the principal cause of cardiovascular disease, stroke, dementia, cancer, non-alcoholic fatty liver disease (NAFLD), and chronic kidney disease (CKD) (2). The latter remains the leading cause of end-stage renal disease (1) and is responsible for the rise in mortality among diabetic patients (3, 4). CKD is characterized by a decrease in glomerular filtration rate, glomerular and tubular hypertrophy, albuminuria, renal inflammation, and tubulo-interstitial fibrosis (5). While current treatments have been shown to delay the progression of the disease and temporarily improve the patient's quality of life, CKD eventually results in dependence on dialysis or kidney transplantation (6). Therefore, there is a critical need to develop more effective, long-lasting treatments for CKD.

Recently, we described a novel series of indoline derivatives with potent antioxidant and anti-inflammatory activities (7). These compounds act by inhibiting p38 MAPK, reducing the degradation of IκBα and the nuclear translocation of NF-κB and AP-1 (8, 9) and have been shown to prevent cellular damage in a mouse model of acute liver disease (10). The aim of the current study was to examine the effect of AN1284 in ameliorating the development of CKD in a murine model of diabesity and its potential to mitigate metabolic abnormalities. Our data show that the compound was able to preserve kidney morphology, ameliorate the development of inflammation and fibrosis, and decrease proteinuria. In addition, AN1284 improved the metabolic profile of the mice, resulting in weight loss via increased fat oxidation, decreased hepatic steatosis, and enhanced insulin sensitivity.

## Materials and Methods

### Animals

The experimental protocols were approved by the Institutional Animal Care and Use Committee of the Hebrew University of Jerusalem (AAALAC accreditation #1285; Ethic approval number MD-17-15302-3) and are in compliance with the ARRIVE guidelines (11). All the animals used in this study were housed under specific pathogen-free conditions, up to five per cage, in standard plastic cages with natural soft sawdust as bedding. The animals were maintained under a controlled temperature of 22–24°C, humidity at 55 ± 5%, and alternating 12-h light/dark cycles and provided with food and water *ad libitum*.

### Induction of Type 2 Diabetes

**Figure 1:**
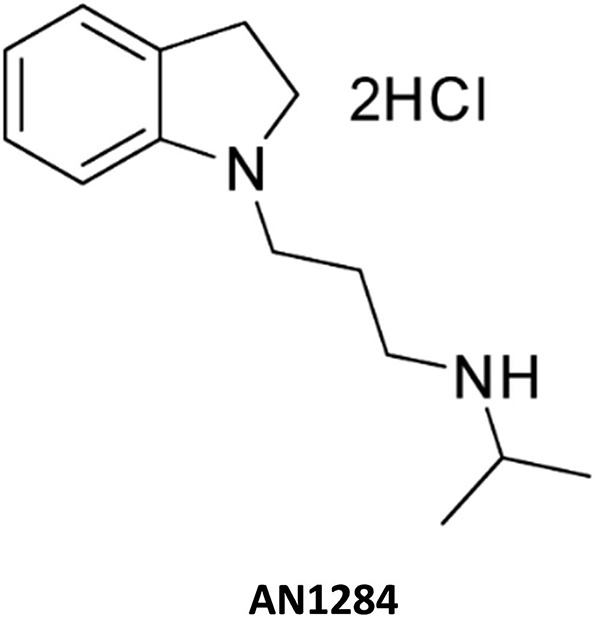
The chemical structure of AN1284.

Male 6-week-old BKS Cg-Dock7^m^+/+Lepr^db^/J mice (BKS *db*; Jackson Laboratory, Cat# 00642) were randomly divided into the experimental groups, 8–10 mice per group, and treated chronically (3 months) with AN1284 [2.5 or 5 mg kg^−1^ per day; Figure 1; prepared as described by Zeeli et al. (7)], by a subcutaneously implanted micro-osmotic pump (Model 1004; alzet®). A new pump was implanted each month. One group of obese BKS.Cg-Dock7^m^ +/+ Lepr^db^/J mice and non-obese controls underwent sham surgery in an identical manner to the treatment groups but without pump insertion. Body weight and glucose levels were monitored weekly. Twenty-four-hour urine output and water consumption were measured using the CCS2000 Chiller System (Hatteras Instruments). Body composition was determined by EchoMRI-100H™ (Echo Medical Systems). Mice were euthanized by cervical dislocation under anesthesia; trunk blood, kidneys, pancreas, liver, muscle, and fat pads were collected, and samples were either snap-frozen or fixed in buffered 4% formalin for further analyses.

### Multi-Parameter Metabolic Assessment

Mice with free access to food and water were housed under a standard 12-h light/12-h dark cycle, which consisted of a 48-h acclimation period followed by 24 h of sampling. Measures of mouse activity and respirometry were made with the Promethion High-Definition Behavioral Phenotyping System (Sable Instruments, Inc., Las Vegas, NV, USA) as described previously (12). Respiratory gases were measured with a GA-3 gas analyzer using a pull-mode, negative-pressure system. Airflow was measured and controlled by FR-8, with a set flow rate of 2,000 ml/min. Water vapor was continuously measured, and its dilution effect on O_2_ and CO_2_ was mathematically compensated. Total energy expenditure (TEE) was calculated as VO_2_ × (3.815 + 1.232 × RQ), normalized to effective body mass, and expressed as kcal/h/kg^0.75^. Fat oxidation (FO) was calculated as FO = 1.69 × VO_2_ – 1.69 × VCO_2_ and expressed as g/d/kg^0.75^. Ambulatory activity and position were monitored simultaneously with the collection of the calorimetry data using XYZ beam arrays with a beam spacing of 0.25 cm. Meal size and total food intake during a period of 24 h were measured automatically by the system.

### Blood, Kidney, and Urine Biochemistry

Serum insulin and urine albumin were measured by ELISA (Millipore and Bethyl Laboratories, respectively). Serum urea, glucose, alanine transaminase (ALT), alkaline phosphatase (ALP), urine glucose and creatinine, total triglycerides, cholesterol, and high- and low-density lipoprotein (HDL and LDL, respectively) were determined using the Cobas C-111 bio-analyzer (Roche, Switzerland). Blood urea nitrogen (BUN) was calculated by serum urea levels (BUN mg/dl = Urea mM × 2.801). Blood glucose was measured with the Elite glucometer (Bayer, Pittsburgh, PA). Glucose and insulin levels were used to calculate the homeostasis model assessment-insulin resistance index (HOMA-IR) as glucose (mg/dl) × insulin (U/L) 22.5. Whole kidney proteins were extracted and the levels of the following markers (MCP1, TNFα, TGFβ, and IL18) were measured using ELISA kits (Abcam; Cat# ab100721, ab46105, ab119557, and ab216165, respectively). Liver proteins were extracted and the levels of TNFα were measured using the same ELISA kit used for the kidney.

### Hepatic Triglyceride and Cholesterol Contents

The liver was extracted as described previously (13), and its triglyceride and cholesterol contents were determined using the Cobas C-111 bio-analyzer (Roche, Switzerland) and normalized to wet tissue weight.

### Histopathology

Paraffin-embedded kidney sections (2 μm) from each mouse were stained with Periodic Acid–Schiff (PAS), followed by hematoxylin. Paraffin-embedded liver sections (5 μm) from each mouse were stained with H&E. Kidney and liver images were taken from 10 random 40× and 20× fields with an AxioCam ICc5 color camera mounted on an Axio Scope.A1 light microscope (Zeiss, Germany). The mesangial expansion, glomerular, and Bowman's space cross-sectional areas were quantified in a blinded manner using ZEN BLUE software (Zeiss, Germany). The hepatic fat area was quantified in the same manner, using Adobe Photoshop CS3 software.

### Immunohistochemistry

Kidney and liver tissues were fixed in buffered 4% formalin for 48 h and then embedded in paraffin. Sections were deparaffinized and hydrated. Heat-mediated antigen was retrieved with 10 mM citrate buffer pH 6.0 (Thermo Scientific, IL, USA). Endogenous peroxide was inhibited by incubating with a freshly prepared 3% H_2_O_2_ solution in MeOH. Unspecific antigens were blocked by incubating sections for 1 h with 2.5% horse serum (VE-S-2000, Vector Laboratories). For assessment of inflammation and fibrosis, the sections were stained with rabbit anti-mouse MCP-1 (Abcam; Cat# ab25124, 1:5,000), IL-18 (Abcam; Cat# ab71495, 1:2,000), TNFα (Abcam; Cat# ab6671, 1:800), TGFβ (Abcam, Cat# ab66043, 1:2,000), Collagen-1 (Abcam; Cat# ab34710, 1:1000), and α-SMA (Abcam; Cat# ab5694, 1:5000) antibodies, followed by incubation with horse anti-rabbit HRP conjugate (MP-7401, ImmPRESS™, Vector laboratories). Color was developed after an incubation with 3,3'-diaminobenzidine (DAB) substrate [ImmPACT DAB Peroxidase (HRP) Substrate, SK-4105, Vector Laboratories], followed by hematoxylin counterstaining and mounting (Vecmount H-5000, Vector laboratories). Stained sections were photographed as mentioned above. Positive areas for each marker were calculated using color thresholding and measuring area fractions by ImageJ software, with a minimum of 10 random kidney and liver sections per mouse. Images are presented in the figures showing the animal with the median value for each group.

For assessing the number of Langerhans β-islets and their composition, pancreatic sections were stained with guinea pig anti-insulin (Dako; Cat# A0564, 1:1,600) antibody, followed by biotinylated secondary antibody and VECTASTAIN ABC reagent (VECTASTAIN ABC-Peroxidase Kit, Vector laboratories). Color was developed as described in the preceding section followed by hematoxylin counterstaining and mounting (Vecmount H-5000, Vector Laboratories). Stained sections were photographed as described above. Panoramic images were taken for the entire section using ZEN BLUE software (Zeiss, Germany) and the islets-to-pancreas ratio and the β-cell-to-islet ratio were calculated.

### Real-Time PCR

Total mRNA from liver and muscle was extracted with Bio-Tri RNA lysis buffer (Bio-Lab, Israel) followed by DNase I treatment (Thermo Scientific, IL, USA) and reverse transcribed using the Qscript cDNA Synthesis Kit (Quanta Biosciences, MA). Real-time PCR was performed using iTaq Universal SYBR Green Supermix (Bio-Rad, CA) and the CFX connect ST system (Bio-Rad, CA), and gene expression quantification was done using the 2∧(-ΔΔCT) method. The primers were as follows: *Col1*α*1* (forward-5′-TTCTCCTGGCAAAGACGGACTCAA-3′, reverse-5′-GGAAGCTGAAGTCATAACCGCCA-3′), *Tgf*β*1* (forward-5′-GCGGACTACTATGCTAAAGAGG-3′, reverse-5′-GTAGAGTTCCACATGTTGCTCC-3′), *Glut4* (forward-5′-ATTTGGGGCCCTAGGTTGTT-3′, reverse-5′-ATACAGCAGCCCTTGGGTTT-3′), *Cxcl10* (forward-5′-GGATGGCTGTCCTAGCTCTG-3′, reverse-5′-TGAGCTAGGGAGGACAAGGA-3′), *Stat6* (forward-5′-AGCCCAGAAACAAAGCCTCTT-3′, reverse-5′-TTCGAGCATTAACACCCCACT-3′), *Klf4* (forward-5′-AGAAGTGTGACAGGGCCTTTT-3′, reverse-5′-TCGTGGGAAGACAGTGTGAAA-3′), *Acc* (forward-5′-GGACCACTGCATGGAATGTTAA-3′, reverse-5′-TGAGTGACTGCCGAAACATCTC-3′). QuantiTect Primer (Qiagen) was used to evaluate the expression of *Scd1* (QT00291151), and *Cd36* (QT0105825). Normalization was performed against *Ubc* (forward-5′-GCCCAGTGTTACCACCAAGA-3′, reverse-5′-CCCATCACACCCAAGAACA-3′) and *Gapdh* (forward-5′-AGGTCGGTGTGAACGGATTTG-3′, reverse-5′-TGTAGACCATGTAGTTGAGGTCA-3′).

### Extraction of AN1284 From Liver and Kidney

Liver and kidney samples from *db/db* mice treated for 3 months with AN1284 were homogenized (100 mg ml^−1^, Polytron, Kinematiea GmbH, Germany) in 1× PBS. To 180 μl of tissue homogenate was added 20 μl of rivastigmine (750 ng ml^−1^) as an internal standard. Proteins were precipitated with 300 μl of ice-cold HPLC-grade MeOH; the mixture was vortexed for 5 min and then allowed to stand for 5 min. This procedure was repeated three times. The samples were centrifuged at 20,800 × *g* at 10°C for 15 min. The supernatant (200 μl) was filtered through 0.25 μM GHP membranes and stored at −80°C until analyzed. Calibration curves for the compounds in liver and kidney homogenates were prepared from untreated mice.

### Quantification of AN1284 by Mass Spectroscopy

Tandem liquid chromatography–mass spectrometry (LC-MS/MS) enables the analysis of complex mixtures and therefore can be applied in pharmacology research. Herein, we analyzed and quantized indoline along with his oxidative major indole metabolite, revealed in the spectrum of the organisms' extractions. A similar indoline-to-indole metabolic conversion has been reported earlier (14). To verify the identity of this metabolite, the compound was prepared as shown in Zeeli et al. (7) (compound 19a).

The targeted molecules were quantified by extracted-ion chromatogram (EIC) of exhibited characteristic parent ions MH^+^ 219.185 and 217.169 m/z, respectively, sharing identical product ion of 132.080.

Analytical system: UHPLC system: Agilent 1260 Technologies consisted of a quaternary pump (G4204A), autosampler (G4226A), column heater (G1316C). System control and data analysis were made by Mass Hunter software. Mass Spectrometer was an Agilent 6545 QTOF LC-MS with “AJS ESI” ion source in ESI + ve mode. Quantitation was performed by monitoring the product ions of the parent molecular ions for the different analytes. LC conditions: column, Poroshell EC-C18, 4.6 × 50 mm, 2.7-micron LC column (Agilent); column temperature, ambient; flow rate, 0.5 ml/min; injection volume, 20 μl; mobile phase, A: water with 0.1% formic acid, B: acetonitrile with 0.1% formic acid.

### Statistics

Values are expressed as the mean ± SEM. Unpaired two-tailed Student's *t* test was used to determine differences between saline and drug-treated groups. Results in multiple groups and time-dependent variables were compared by ANOVA, followed by Tukey's *post hoc* analysis (GraphPad Prism v6 for Windows). Significance was set at *P* < 0.05.

## Results

### AN1284 Preserves Kidney Morphology and Function in Type 2 Diabetic Mice

**Figure 2:**
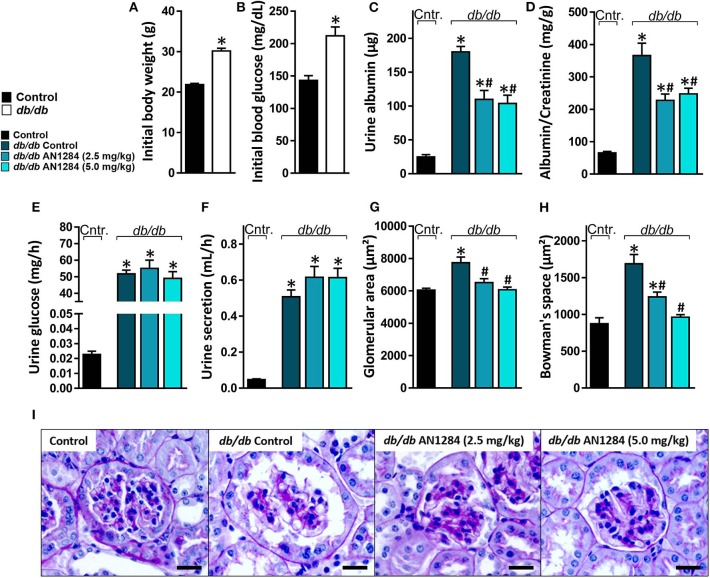
AN1284 preserves kidney morphology and function in type 2 diabetic mice. Compared to the healthy controls, *db/db* mice exhibited increased body weight **(A)** and hyperglycemia **(B)** at the beginning of the experiment. AN1284 significantly reduced the elevated levels of urine albumin **(C)** and the albumin-to-creatinine ratio **(D)**, but not the urine glucose levels **(E)** and the urine secretion volume **(F)**. AN1284-treated *db/db* animals exhibited a reduction in the glomerular area **(G,I)**, and in Bowman's space area **(H,I)**. Scale bar, 20 μm. Data represent the mean ± SEM from 8 to 10 mice per group. *P < 0.05 relative to control non-diabetic mice, ^#^*P* < 0.05 relative to *db/db*-Veh-treated control mice.

**Figure 3:**
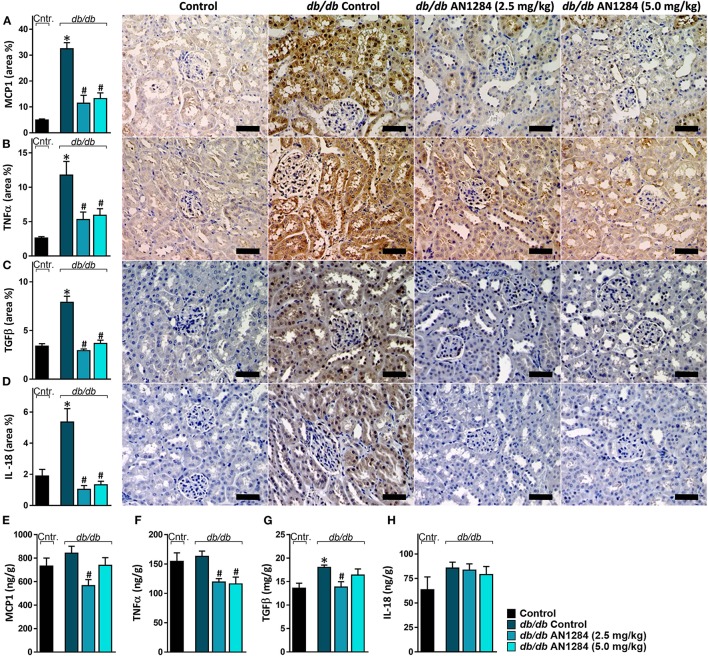
AN1284 prevents the renal inflammatory response in type 2 diabetic mice. The elevated renal protein expression levels of the inflammatory markers MCP-1 **(A,E)**, TNFα **(B,F)**, TGFβ **(C,G)**, and IL-18 **(D,H)** in *db/db* mice were normalized by AN1284 treatment. Scale bar, 50 μm. Data represent the mean ± SEM from 8 to 10 mice per group. *P < 0.05 relative to control non-diabetic mice, ^#^*P* < 0.05 relative to *db/db*-Veh-treated control mice.

At the beginning of the experiment, the *db/db* mice exhibited higher body weight and blood glucose levels than did their healthy controls (Figures 2A,B), indicating an early onset of obesity and type 2 diabetes, which further progressed during the experiment. At the end of 3 months, the diabetic mice developed complete CKD, manifested by renal dysfunction and morphology. AN1284 treatment lessened the renal damage, as seen by a reduction in albuminuria and albumin-to-creatinine (ACR) ratio (Figures 2C,D). AN1284 treatment prevented the severe glomerular enlargement and that of Bowman's capsule in the diabetic mice (Figures 2G–I), but not the rise in urine glucose levels (Figure 2E) or the polyuria (Figure 2F). We showed that type 2 diabetes in this model is associated with renal inflammation, as indicated by the increased protein expression of inflammatory markers MCP1, TNFα, TGFβ, and IL-18. This up-regulation was significantly reduced by AN1284 (Figures 3A–H).

### AN1284 Mitigates the Increased Body Weight via Increasing Fat Oxidation

**Figure 4:**
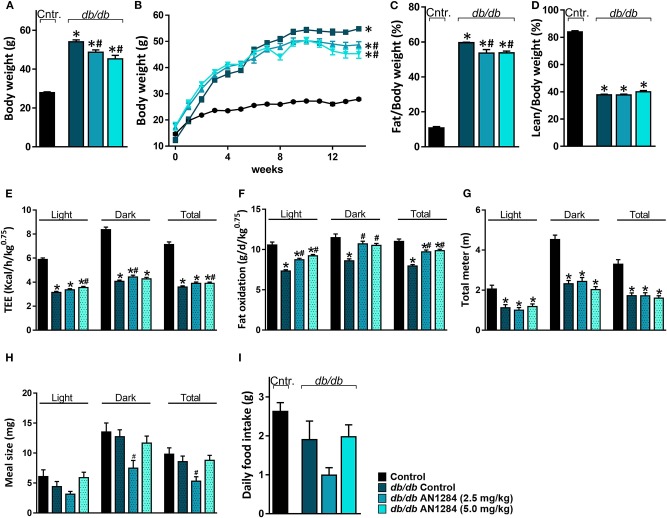
AN1284 mitigates increased body weight in *db/db* mice via increasing fat oxidation. Chronic treatment with AN1284 significantly attenuated the increased body weight **(A,B)** and fat mass **(C)** without affecting lean mass **(D)** in *db/db* mice. Significant increases in total energy expenditure (TEE; **E**) and fat oxidation **(F)** were measured in *db/db* mice chronically treated with AN1284. No effect was found on mouse activity as measured by the total distance the mice moved in their cages **(G)**. *db/db* mice treated with the lower dose of AN1284 exhibited decreased meal size **(H)**, without a significant effect on daily food intake **(I)**. Data represent the mean ± SEM from 8 to 10 mice per group. *P < 0.05 relative to control non-diabetic mice, ^#^*P* < 0.05 relative to *db/db*-Veh-treated control mice.

Although AN1284-treated mice were significantly heavier than the normal controls, they did not reach the same level of obesity as the untreated BSK *db* mice (Figure 4A) and their weight no longer increased after 8 weeks of treatment (Figure 4B). As a result, analysis of body composition confirmed that the arrested weight gain is due to a lower body fat accumulation with no change in lean body mass (Figures 4C,D). Full metabolic assessment revealed that AN1284 increased TEE due to increased fat oxidation during both the light and dark periods without affecting the animals' activity (Figures 4E–G). Interestingly, meal size was significantly reduced in *db/db* mice treated with the lower dose of AN1284 (Figure 4H); however, it did not significantly affect total daily food intake measured while the animals were housed in the metabolic chambers for 24 h (Figure 4I).

### AN1284 Increases Insulin Sensitivity

**Figure 5:**
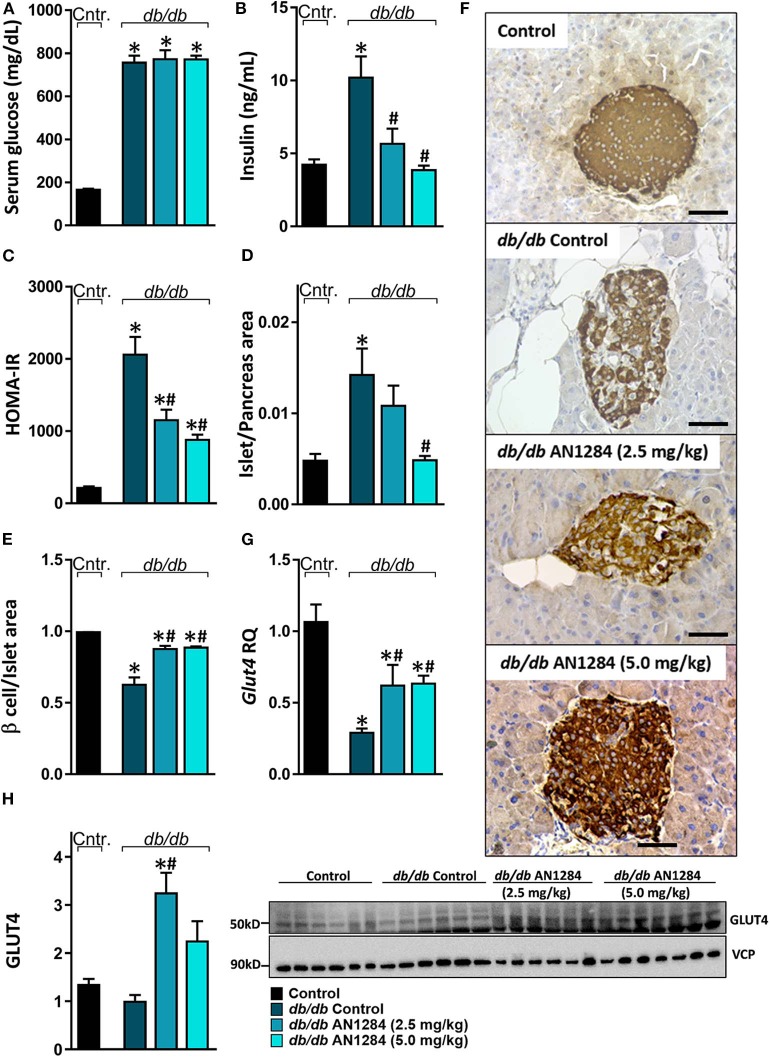
AN1284 increases insulin sensitivity in *db/db* mice. The *db/db* mice exhibit hyperglycemia and hyperinsulinemia (**A,B**, respectively), compared with healthy controls. AN1284 treatment significantly reduced serum insulin levels without changing those of serum glucose. A reduced HOMA-IR **(C)** and Langerhans islet-to-pancreas tissue ratio **(D)**, and an increased β cell-to-Langerhans islet area **(E)** and *Glut4* mRNA **(G)** as well as protein expression **(H)** were found in *db/db* mice treated with AN1284. **(F)** Representative insulin staining of the pancreas from each treatment group. Original magnification, ×40. Scale bar, 50 mm. Data represent the mean ± SEM from 8 to 10 mice per group. *P < 0.05 relative to control non-diabetic mice, ^#^*P* < 0.05 relative to *db/db*-Veh-treated control mice.

All the *db/db* mice displayed extreme hyperglycemia (Figure 5A), but a reduction in hyperinsulinemia and HOMA-IR was found in the AN1284-treated groups (Figures 5B,C). Histological analysis of the pancreas revealed an increase in the area of the Langerhans islet in the untreated BSK *db* mice. This was completely normalized by treatment with the higher dose of AN1284 (Figure 5D). A diabetic pancreas also undergoes massive β cell degeneration (15). AN1284-treated mice exhibited a higher percentage of functional β cells and the ability to secrete insulin than the untreated animals (Figures 5E,F). One reason for the development of a loss of insulin sensitivity is the downregulation of expression of the GLUT4 transporter, as shown in *db/db* mice (16). This was demonstrated in the skeletal muscle of the untreated diabetic mice, and GLUT4 mRNA and protein expression levels were improved by AN1284 treatment (Figures 5G,H). Taken together, these findings suggest that AN1284 has the ability to increase insulin sensitivity.

### AN1284 Ameliorates Hepatic Steatosis, Liver Injury, and Fibrosis

**Figure 6:**
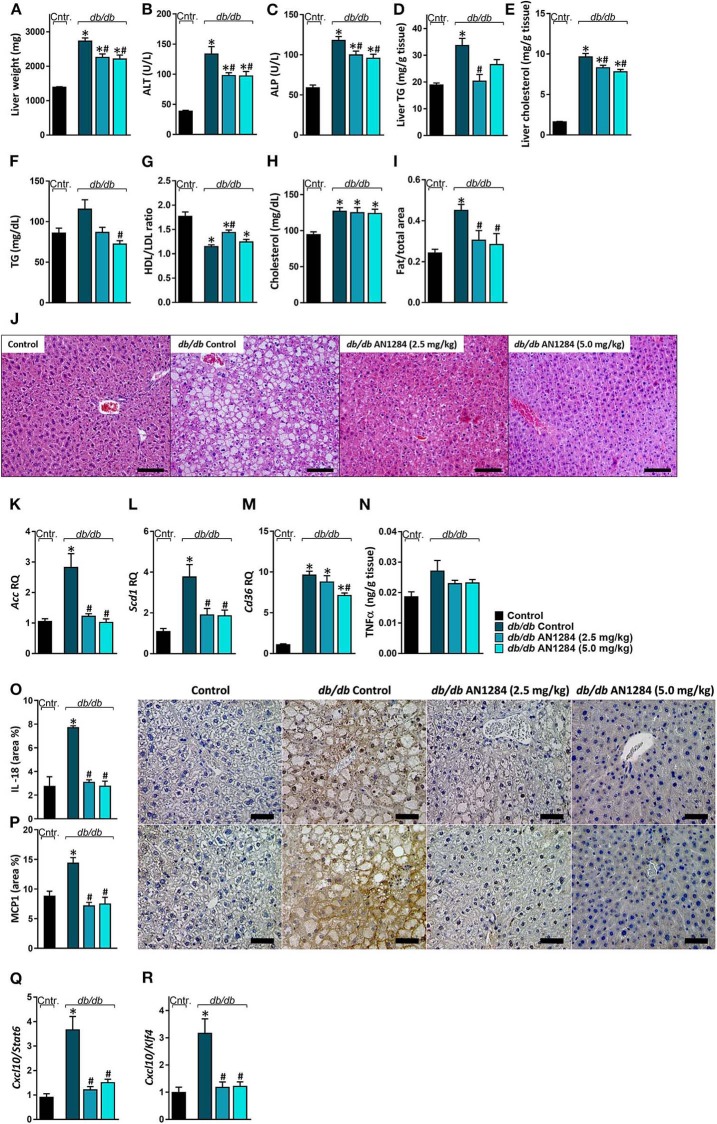
AN1284 ameliorates hepatic steatosis and inflammation in *db/db* mice. The *db/db* mice exhibit increased liver weight **(A)**, serum ALT **(B)**, and ALP **(C)** levels as well as hepatic triglyceride and cholesterol contents **(D,E)**. These parameters were prevented or ameliorated by AN1284 treatment. AN1284-treated mice also displayed decreased serum triglyceride levels **(F)**, increased HDL/LDL cholesterol ratio **(G)**, without an effect on total cholesterol levels **(H)**. Quantification of hepatic fat content revealed an increase in *db/db* animals, which was normalized by AN1284 treatment **(I)**. Representative liver images demonstrating macrovesicular steatosis in H&E-stained sections from *db/db* mice. Scale bar, 100 μm **(J)**. The elevated hepatic mRNA expression levels of *Acc* **(K)**, *Scd1* **(L)**, and *Cd36* **(M)** in *db/db* mice were normalized by AN1284 treatment. Insignificant changes in hepatic TNFα protein levels were noted in non-treated and treated mice **(N)**. Reduced protein expression of IL-18 **(O)**, and MCP1 **(P)** in AN1284-treated *db/db* mice was documented. Scale bar, 50 μm. M1-to-M2 macrophage marker ratios displayed high content of M1 macrophages in *db/db* animals, which was diminished by AN1284 treatment **(Q,R)**. Data represent the mean ± SEM from 8 to 10 mice per group. *P < 0.05 relative to control non-diabetic mice, ^#^*P* < 0.05 relative to *db/db*-Veh-treated control mice.

**Figure 7:**
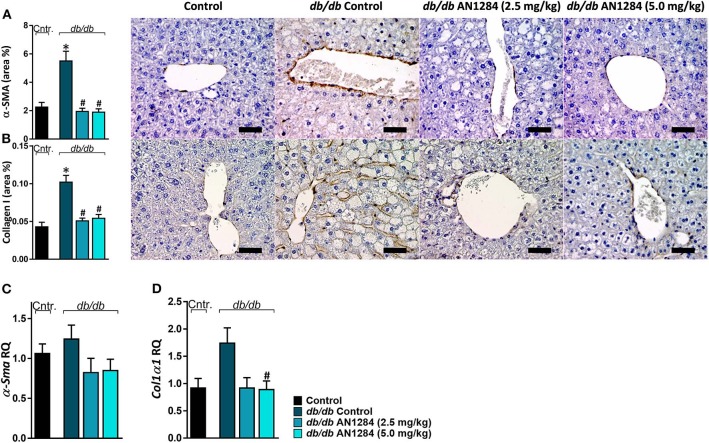
AN1284 mitigates hepatic fibrosis in *db/db* mice. Untreated *db/db* animals exhibited increased protein and mRNA expression levels of the fibrotic markers α-SMA **(A,C)** and Collagen I **(B,D)**, which were normalized in AN1284-treated mice. Scale bar, 50 μm. Data represent the mean ± SEM from 8 to 10 mice per group. *P < 0.05 relative to control non-diabetic mice, ^#^*P* < 0.05 relative to *db/db*-Veh-treated control mice.

AN1284 was able to decrease hepatocellular damage and hepatic steatosis, as manifested by a lower liver weight (Figure 6A), serum levels of ALT and ALP (Figures 6B,C), as well as hepatic triglyceride and cholesterol contents (Figures 6D,E). AN1284 reduced serum triglyceride levels and increased HDL/LDL ratio, but did not affect total serum cholesterol (Figures 6F–H). AN1284-treated *db/db* mice showed a reduced accumulation of fat in hepatocytes (Figures 6I,J). The latter was most likely mediated through the ability of AN1284 to downregulate the hepatic expression of several key genes involved in *de novo* lipogenesis (*Acc* and *Scd1*; Figures 6K,L, respectively) and fatty acids transport (*Cd36*; Figure 6M). Although only a trend toward reduction in hepatic TNFα levels was noted in *db/db* mice treated with AN1284 (Figure 6N), the levels of protein expression of IL-18 and MCP1 were significantly decreased (Figures 6O,P). This effect is most likely mediated by the ability of AN1284 to induce a shift in macrophages toward a predominantly M2 phenotype (Figures 6Q,R). Moreover, AN1284 also reduced the elevated mRNA and protein expression of the hepatic fibrogenic markers, α-SMA, and Collagen 1 (Figures 7A–D), suggesting that AN1284 has anti-fibrogenic activity in the liver.

## Discussion

Obesity commonly precedes type 2 diabetes and results in the metabolic syndrome and hypertension, which increases pressure on the kidney. Following weight gain, hyper-filtration develops to meet the increased metabolic demand (17). About half of the patients with type 2 diabetes and one third of those with type 1 diabetes develop nephropathy (18, 19). Although the molecular mechanisms contributing to the development of CKD in diabetes and obesity are quite different, it is accompanied in each condition by glomerular hypertrophy and transient hyper-filtration, glomerular basement membrane thickening, and mesangial matrix expansion, resulting in albuminuria (20, 21). Together with renal inflammation (22) and oxidative stress (23), these changes may lead to renal fibrosis, and ultimately to a progressive decline in glomerular filtration rate (5). Normalization of blood pressure and glucose levels can protect the kidneys and reduce the likelihood of developing CKD. However, a significant number of patients with diabetes may still progress to end-stage renal disease (24), stressing the importance of developing novel drugs to treat CKD. Here, we show that the indoline derivative, AN1284, has a therapeutic potential to ameliorate CKD in type 2 diabetic conditions in mice. The *db/db* mouse model of type 2 diabetes was chosen for this study rather than a high-fat diet-induced model of metabolic disorder because we were primarily interested in trying to prevent the development of CKD, which is greater in *db/db* mice due to hyperglycemia and hyperlipidemia.

We have previously reported that a series of novel indoline derivatives possess antioxidant and anti-inflammatory properties (7, 8). The therapeutic potential of these novel molecules (with or without a carbamate moiety) was tested in murine models of acute lung injury (9), ulcerative colitis (25), and acute liver injury (10). The current study demonstrates that one of them, AN1284, is also effective in mitigating the development of diabetes-induced CKD, which is a chronic condition. Although we do not know the identity of specific targets activated by AN1284, we previously reported that AN1284 reduced the phosphorylation of p38 MAP kinase and the nuclear translocation of activator protein 1 in LPS-activated macrophages and in the liver of mice with acute liver injury (10). We are currently performing RNA seq analysis and high-throughput bioassay screening including *in silico* and data-mining methodologies to try to decipher specific targets for AN1284. Here, we administered AN1284 by subcutaneously implanting mini-pumps for 12 weeks, and the treatment was started after confirming the presence of hyperglycemia. Although the drug was unable to reduce blood glucose levels, it improved renal function and its histopathological appearance and reduced renal inflammatory and fibrogenic markers. The concentrations of the drug found in the kidney and liver (Table 1) were sufficient to cause an anti-inflammatory and antioxidant effect (7). AN1284 was very effective in reducing renal inflammation in the kidney at both doses tested here. The higher dose of AN1284 caused significantly greater polyuria than that seen in the untreated mice. It is possible that the added diuretic effect is mediated by another mechanism, such as inhibition of sodium reabsorption in the renal tubule cells, which is also seen in human subjects given Indapamide (26) that also contains an indoline group. However, AN1284 lacks the thiazide group of Indapamide and of other potent diuretics, making this less likely.

**Table 1:** Concentration of AN1284 and its metabolite in liver and kidney.

**Dose (mg kg^**−1**^ per day)**	**Concentration** **±** **STD (ng/g)**			
	**Kidney**		**Liver**	
	**AN1284**	**Metabolite**	**AN1284**	**Metabolite**
2.5	140 ± 69	116 ± 30	36.2 ± 7.3	13.7 ± 2.7
5.0	298 ± 52	181 ± 17	40.5 ± 3.7	17.0 ± 5.6

Other current therapies for attenuating the progression of diabesity-induced CKD focus on ameliorating risk factors, such as visceral obesity, insulin resistance, and NAFLD. Evidence shows that lifestyle modifications resulting in weight loss, bariatric surgery, and pharmacotherapy may arrest renal dysfunction in patients with type 2 diabetes [reviewed in Docherty et al. (27)]. Moreover, a modest weight loss has been reported to decrease proteinuria in such patients (28). We also observed a significant body weight loss, together with up to 11% fat mass loss in the AN1284-treated mice. These effects can be explained by the increased TEE and fat oxidation, and not by drug-induced changes in the activity of the animals.

Insulin resistance, assessed by HOMA-IR, is present in patients with mild to severe CKD (29). In a few cross-sectional studies in humans, insulin resistance has been linked to the risk of developing diabetes-induced CKD (30, 31). On a molecular basis, both the direct and indirect effects of hyperinsulinemia play a key role in kidney function. Under type 2 diabetic conditions, pancreatic islets respond not only by increasing insulin secretion but also by expanding β cell mass (32). The latter was completely normalized by treatment with the AN1284 (5 mg kg^−1^ per day). Hyperinsulinemia *per se* may induce glomerular hyperfiltration, endothelial dysfunction, and increased vascular permeability, which together may lead to albuminuria (33). In addition, insulin resistance is associated with altered renal cellular metabolism, mesangial expansion, renal inflammation, and lipid deposition in the renal proximal tubule cells, all established contributors to nephropathy (34). Together with the critical role of insulin in secreting different adipokines and chemokines, which are known to modulate kidney function, increased insulin sensitivity may attenuate the development and progression of CKD. Tejada and colleagues have shown that podocytes isolated from *db/db* mice are unable to respond to insulin (35). Tiwari et al. reported downregulation of the renal insulin receptor in several type 2 diabetes models (36), and that its complete deletion in the proximal tubule cells results in albuminuria (37). AN1284 may improve renal function by reducing insulin levels and the increase in pancreatic β-cell mass in *db/db* mice. Thus, AN1284 reversed renal insulin resistance by decreasing the direct effect of insulin on podocytes or tubular cells, which express all elements of the insulin-signaling cascade (38). Nevertheless, as the ability of AN1284 to reverse insulin sensitivity directly was not fully characterized here (by assessing whole-body glucose utilization in live animals), we cannot conclude whether AN1284 suppresses hepatic glucose production or improves skeletal or white adipose tissue insulin sensitivity to improve HOMA-IR reported here. These issues as well as the underlying molecular mechanisms by which AN1284 ameliorates insulin tolerance are important questions that will need to be addressed in the near future.

Several lines of evidence suggest that NAFLD promotes type 2 diabetes. While NAFLD is present in 20–30% of the general population (39), it reaches a prevalence of 50–75% of patients affected by diabesity (40). Conversely, insulin resistance, which occurs in 66–83% of patients with NAFLD, increases the flux of free fatty acids from adipocytes to the liver and promotes hepatic lipid accumulation and liver injury. Once diabesity is fully developed, it further contributes to the development of steatosis and also to hepatic inflammation and fibrosis (41). To date, there is evidence linking NAFLD, as a causative player, to the development and progression of CKD (42). Furthermore, several large cross-sectional studies of patients with NAFLD show a prevalence of CKD between 4 and 40% and a positive correlation between the severity of NALFD and CKD [reviewed in (43)]. Whereas, the complex “crosstalk” among adipose tissue, the liver, and kidneys makes it difficult to decipher the specific pathway underlying NAFLD as a cause of CKD, it is not surprising that these diseases may be linked, and therefore the amelioration of NAFLD may also contribute to the reversal of CKD. Importantly, hepatic steatosis, liver injury, dyslipidemia, inflammation, and fibrosis present in *db/db* mice were significantly ameliorated by chronic treatment with AN1284. With its ability to mitigate acute liver injury via reduced inflammation (10), our current data strongly support the idea that an anti-inflammatory agent like AN1284 could provide a therapeutic intervention for the treatment of NAFLD and may also assist in the prevention of diabesity-induced CKD. At present, we cannot differentiate between direct or indirect effects of AN1284 since no specific target(s) have been identified. AN1284 positively affects kidney, liver, muscle, and pancreatic functions. Based on the very marginal effect that AN1284 had on body weight (the animals still remained obese), we can only postulate that the improvements in multiple processes may be mediated via a general anti-inflammatory effect and specific actions of AN1284 on various renal cells (podocytes, tubular, and mesangial cells), hepatocytes, myocytes, and pancreatic beta cells. Further work is needed to be done to understand the molecular mechanism(s) by which AN1284 improves diabesity-induced CKD.

## Data Availability Statement

The datasets generated for this study can be found in the https://patents.google.com/patent/WO2017125932A1/en.

## Ethics Statement

The experimental protocols were approved by the Institutional Animal Care and Use Committee of the Hebrew University of Jerusalem (AAALAC accreditation #1285; Ethic approval number MD-17-15302-3).

## Author Contributions

AP conducted the experiments and analyzed the data. AG and LH assisted in experiments. MWeit conducted the LC-MS/MS analysis. MWein and JT designed and supervised the experiments, and analyzed the data. AP, MWein, and JT wrote the manuscript.

### Conflict of Interest

The authors declare that the research was conducted in the absence of any commercial or financial relationships that could be construed as a potential conflict of interest.

## References

[1] Farag YM Gaballa MR. Diabesity: an overview of a rising epidemic. Nephrol Dial Transplant. (2011) 26:28–35. 10.1093/ndt/gfq576 21045078

[2] Martinez Leo EE Acevedo Fernandez JJ Segura Campos MR. Biopeptides with antioxidant and anti-inflammatory potential in the prevention and treatment of diabesity disease. Biomed Pharmacother. (2016) 83:816–26. 10.1016/j.biopha.2016.07.051 27501499

[3] Bruno G Merletti F Bargero G Novelli G Melis D Soddu A. Estimated glomerular filtration rate, albuminuria and mortality in type 2 diabetes: the Casale Monferrato study. Diabetologia. (2007) 50:941–8. 10.1007/s00125-007-0616-1 17333106

[4] Afkarian M Sachs MC Kestenbaum B Hirsch IB Tuttle KR Himmelfarb J. Kidney disease and increased mortality risk in type 2 diabetes. J Am Soc Nephrol. (2013) 24:302–8. 10.1681/ASN.2012070718 23362314 PMC3559486

[5] Decleves AE Sharma K. New pharmacological treatments for improving renal outcomes in diabetes. Nat Rev Nephrol. (2010) 6:371–80. 10.1038/nrneph.2010.57 20440278

[6] Braun L Sood V Hogue S Lieberman B Copley-Merriman C. High burden and unmet patient needs in chronic kidney disease. Int J Nephrol Renovasc Dis. (2012) 5:151–63. 10.2147/IJNRD.S37766 23293534 PMC3534533

[7] Zeeli S Weill T Finkin-Groner E Bejar C Melamed M Furman S. Synthesis and biological evaluation of derivatives of indoline as highly potent antioxidant and anti-inflammatory agents. J Med Chem. (2018) 61:4004–19. 10.1021/acs.jmedchem.8b00001 29681148

[8] Furman S Nissim-Bardugo E Zeeli S Weitman M Nudelman A Finkin-Groner E. Synthesis and in vitro evaluation of anti-inflammatory activity of ester and amine derivatives of indoline in RAW 264.7 and peritoneal macrophages. Bioorg Med Chem Lett. (2014) 24:2283–7. 10.1016/j.bmcl.2014.03.081 24731278

[9] Finkin-Groner E Moradov D Shifrin H Bejar C Nudelman A Weinstock M. Indoline-3-propionate and 3-aminopropyl carbamates reduce lung injury and pro-inflammatory cytokines induced in mice by LPS. Br J Pharmacol. (2015) 172:1101–13. 10.1111/bph.12982 25322956 PMC4314198

[10] Finkin-Groner E Finkin S Zeeli S Weinstock M. Indoline derivatives mitigate liver damage in a mouse model of acute liver injury. Pharmacol Rep. (2017) 69:894–902. 10.1016/j.pharep.2017.03.025 28628850

[11] Kilkenny C Browne W Cuthill IC Emerson M Altman DG NC3Rs Reporting Guidelines Working Group. Animal research: reporting in vivo experiments: the ARRIVE guidelines. Br J Pharmacol. (2010) 160:1577–9. 10.1111/j.1476-5381.2010.00872.x 20649561 PMC2936830

[12] Udi S Hinden L Earley B Drori A Reuveni N Hadar R. Proximal tubular cannabinoid-1 receptor regulates obesity-induced CKD. J Am Soc Nephrol. (2017) 28:3518–32. 10.1681/ASN.2016101085 28860163 PMC5698062

[13] Folch J Lees M Sloane Stanley GH. A simple method for the isolation and purification of total lipides from animal tissues. J Biol Chem. (1957) 226:497–509. 13428781

[14] Sun H Ehlhardt WJ Kulanthaivel P Lanza DL Reilly CA Yost GS. Dehydrogenation of indoline by cytochrome P450 enzymes: a novel “aromatase” process. J Pharmacol Exp Ther. (2007) 322:843–51. 10.1124/jpet.107.121723 17502430

[15] Gerber PA Rutter GA. The role of oxidative stress and hypoxia in pancreatic beta-cell dysfunction in diabetes mellitus. Antioxid Redox Signal. (2017) 26:501–18. 10.1089/ars.2016.6755 27225690 PMC5372767

[16] Kang OH Shon MY Kong R Seo YS Zhou T Kim DY. Anti-diabetic effect of black ginseng extract by augmentation of AMPK protein activity and upregulation of GLUT2 and GLUT4 expression in db/db mice. BMC Complement Altern Med. (2017) 17:341. 10.1186/s12906-017-1839-4 28662663 PMC5492680

[17] Kovesdy CP Furth S Zoccali C. Obesity and kidney disease: hidden consequences of the epidemic. Rev Med Chil. (2017) 145:281–91. 10.4067/S0034-98872017000300001 28548184

[18] Anders HJ Huber TB Isermann B Schiffer M. CKD in diabetes: diabetic kidney disease versus nondiabetic kidney disease. Nat Rev Nephrol. (2018) 14:361–77. 10.1038/s41581-018-0001-y 29654297

[19] Sulaiman MK. Diabetic nephropathy: recent advances in pathophysiology and challenges in dietary management. Diabetol Metab Syndr. (2019) 11:7. 10.1186/s13098-019-0403-4 30679960 PMC6343294

[20] Ogden CL Yanovski SZ Carroll MD Flegal KM. The epidemiology of obesity. Gastroenterology. (2007) 132:2087–102. 10.1053/j.gastro.2007.03.052 17498505

[21] Kramer H Luke A Bidani A Cao G Cooper R McGee D. Obesity and prevalent and incident CKD: the Hypertension Detection and Follow-Up Program. Am J Kidney Dis. (2005) 46:587–94. 10.1053/j.ajkd.2005.06.007 16183412

[22] Stemmer K Perez-Tilve D Ananthakrishnan G Bort A Seeley RJ Tschop MH. High-fat-diet-induced obesity causes an inflammatory and tumor-promoting microenvironment in the rat kidney. Dis Model Mech. (2012) 5:627–35. 10.1242/dmm.009407 22422828 PMC3424460

[23] Lee W Eom DW Jung Y Yamabe N Lee S Jeon Y. Dendrobium moniliforme attenuates high-fat diet-induced renal damage in mice through the regulation of lipid-induced oxidative stress. Am J Chin Med. (2012) 40:1217–28. 10.1142/S0192415X12500905 23227793

[24] Kim MK. Treatment of diabetic kidney disease: current and future targets. Korean J Intern Med. (2017) 32:622–30. 10.3904/kjim.2016.219 28704915 PMC5511942

[25] Shifrin H Moradov D Bejar C Schorer-Apelbaum D Weinstock M. Novel indoline derivatives prevent inflammation and ulceration in dinitro-benzene sulfonic acid-induced colitis in rats. Pharmacol Rep. (2016) 68:1312–8. 10.1016/j.pharep.2016.08.008 27710861

[26] Pruss T Wolf PS. Preclinical studies of indapamide, a new 2-methylindoline antihypertensive diuretic. Am Heart J. (1983) 106(1 Pt 2):208–11. 10.1016/0002-8703(83)90118-7 6869202

[27] Docherty NG Canney AL le Roux CW. Weight loss interventions and progression of diabetic kidney disease. Curr Diab Rep. (2015) 15:55. 10.1007/s11892-015-0625-2 26122095

[28] Praga M Morales E. Weight loss and proteinuria. Contrib Nephrol. (2006) 151:221–9. 10.1159/000095332 16929145

[29] Svensson M Eriksson JW. Insulin resistance in diabetic nephropathy–cause or consequence? Diabetes Metab Res Rev. (2006) 22:401–10. 10.1002/dmrr.648 16703644

[30] De Cosmo S Trevisan R Minenna A Vedovato M Viti R Santini SA. Insulin resistance and the cluster of abnormalities related to the metabolic syndrome are associated with reduced glomerular filtration rate in patients with type 2 diabetes. Diabetes Care. (2006) 29:432–4. 10.2337/diacare.29.02.06.dc05-1841 16443904

[31] Jauregui A Mintz DH Mundel P Fornoni A. Role of altered insulin signaling pathways in the pathogenesis of podocyte malfunction and microalbuminuria. Curr Opin Nephrol Hypertens. (2009) 18:539–45. 10.1097/MNH.0b013e32832f7002 19724224 PMC2907246

[32] Prentki M Nolan CJ. Islet beta cell failure in type 2 diabetes. J Clin Invest. (2006) 116:1802–12. 10.1172/JCI29103 16823478 PMC1483155

[33] Groop PH Forsblom C Thomas MC. Mechanisms of disease: Pathway-selective insulin resistance and microvascular complications of diabetes. Nat Clin Pract Endocrinol Metab. (2005) 1:100–10. 10.1038/ncpendmet0046 16929378

[34] De Cosmo S Menzaghi C Prudente S Trischitta V. Role of insulin resistance in kidney dysfunction: insights into the mechanism and epidemiological evidence. Nephrol Dial Transplant. (2013) 28:29–36. 10.1093/ndt/gfs290 23048172

[35] Tejada T Catanuto P Ijaz A Santos JV Xia X Sanchez P. Failure to phosphorylate AKT in podocytes from mice with early diabetic nephropathy promotes cell death. Kidney Int. (2008) 73:1385–93. 10.1038/ki.2008.109 18385666

[36] Tiwari S Halagappa VK Riazi S Hu X Ecelbarger CA. Reduced expression of insulin receptors in the kidneys of insulin-resistant rats. J Am Soc Nephrol. (2007) 18:2661–71. 10.1681/ASN.2006121410 17855644

[37] Kumari M Sharma R Pandey G Ecelbarger CM Mishra P Tiwari S. Deletion of insulin receptor in the proximal tubule and fasting augment albumin excretion. J Cell Biochem. (2019) 120:10688–96. 10.1002/jcb.28359 30644120

[38] Horita S Nakamura M Suzuki M Satoh N Suzuki A Seki G. Selective insulin resistance in the kidney. Biomed Res Int. (2016) 2016:5825170. 10.1155/2016/5825170 27247938 PMC4876201

[39] de Alwis NM Day CP. Non-alcoholic fatty liver disease: the mist gradually clears. J Hepatol. (2008) 48:S104–S112. 10.1016/j.jhep.2008.01.009 18304679

[40] Gupte P Amarapurkar D Agal S Baijal R Kulshrestha P Pramanik S. Non-alcoholic steatohepatitis in type 2 diabetes mellitus. J Gastroenterol Hepatol. (2004) 19:854–8. 10.1111/j.1440-1746.2004.03312.x 15242486

[41] Smith BW Adams LA. Nonalcoholic fatty liver disease and diabetes mellitus: pathogenesis and treatment. Nat Rev Endocrinol. (2011) 7:456–65. 10.1038/nrendo.2011.72 21556019

[42] Loomba R Sanyal AJ. The global NAFLD epidemic. Nat Rev Gastroenterol Hepatol. (2013) 10:686–90. 10.1038/nrgastro.2013.171 24042449

[43] Marcuccilli M Chonchol M. NAFLD and chronic kidney disease. Int J Mol Sci. (2016) 17:562. 10.3390/ijms17040562 27089331 PMC4849018

